# Heterologous production of hyaluronic acid in *Nicotiana tabacum* hairy roots expressing a human hyaluronan synthase 2

**DOI:** 10.1038/s41598-021-97139-0

**Published:** 2021-09-09

**Authors:** Arezoo Nazeri, Ali Niazi, Alireza Afsharifar, Seyed Mohsen Taghavi, Ali Moghadam, Farzaneh Aram

**Affiliations:** 1 Institute of Biotechnology, Shiraz University, Shiraz, Iran; 2 Plant Virology Research Center, College of Agriculture, Shiraz University, Shiraz, Iran; 3 Department of Plant Protection, College of Agriculture, Shiraz University, Shiraz, Iran

**Keywords:** Biotechnology, Plant sciences

## Abstract

Hyaluronic acid (HA), a unique polysaccharide with excellent Physico-chemical properties, is broadly used in pharmaceutical, biomedical, and cosmetic fields. It is widely present in all vertebrates, certain bacterial strains, and even viruses while it is not found in plants, fungi, and insects. HA is naturally synthesized by a class of integral membrane proteins called Hyaluronic acid synthase (HAS). Thus far, industrial production of HA is carried out based on either extraction from animal sources or large-scale microbial fermentation. The major drawbacks to using these systems are contamination with pathogens and microbial toxins. Recently, the production of HA through recombinant systems has received considerable attention. Plants are eco-friendly ideal expression systems for biopharmaceuticals production. In this study, the optimized human hyaluronic acid synthase2 (*hHAS2*) sequence was transformed into *Nicotiana tabacum* using *Agrobacterium rhizogenes*. The highest rhHAS2 concentration of 65.72 ng/kg (wet weight) in transgenic tobacco hairy roots was measured by the human HAS2 ELISA kit. The HA production in the transgenic hairy roots was verified by scanning electron microscope (SEM) and quantified by the HA ELISA kit. The DPPH radical scavenging activity of HA with the highest concentration of 0.56 g/kg (wet weight) showed a maximum activity of 46%. Gel Permeation Chromatography (GPC) analyses revealed the high molecular weight HA (HMW-HA) with about > 0.8 MDa.

## Introduction

Hyaluronic acid (HA) (also called Hyaluronan or Hyaluronate), a linear polyanionic mucopolysaccharide, is classified as a Glycosaminoglycan (GAG)^1^. It is composed of repeating disaccharide subunits of *N*-acetyl-d-glucosamine and d-glucuronic acid connected by β-1,3-glycosidic bonds, in a way that each repeating disaccharide unit is connected to the other by β-1,4-glycosidic bonds^2^. Hyaluronan is a very interesting molecule famous for its unique Physico-chemical properties^4^. Several studies have indicated that HA is involved in cell signaling, wound repair, tissue regeneration, morphogenesis, matrix organization, embryogenesis, inflammation, and angiogenesis^3^. HA provides a broad range of medical^4,5^, pharmaceutical^6^, cosmetic^7,8^, and dietary^9^ applications. Due to its application in various fields, it is predicted that the HA global market will reach approximately U$D 15.25 Billion by 2026^10^. Hyaluronan is synthesized by an integral membrane protein known as Hyaluronic acid synthase (HAS)^11^. HAS enzymes are coupled to cell membranes of organisms and synthesize large polymers of the repeating disaccharide structure of HA by alternate addition of UDP-glucuronic acid and UDP-N-Acetylglucosamine moieties to the growing chains^12^. In the human body, HA is synthesized by three HAS isoenzymes (HAS1, HAS2, and HAS3) that are expressed in different tissues^9^. These isoenzymes differ in kinetic characteristics, the half-life of activity, and product size^13^. HAS1 is the least active isoenzyme with the lowest rate of synthesis of HA and quickly loses its activity^11^. HAS2 is the main hyaluronan synthetic enzyme for human survival, which synthesizes long HA chains^8,9^. HAS1 and HAS2 produce large-sized hyaluronic acid. HAS3 is the most active HAS isoenzyme synthesizing HA with low molecular weight in specific conditions^11^.

HA is widely found in nature for instance in vertebrates^14^, the capsular component of certain bacteria^15^, the green algae Chlorella sp. affected by the *Chlorovirus*^14,16^, yeasts^14^, and mollusks^17^. However, it is not found in plants, insects, and fungi^8^.

Thus far, HA has been extracted from animal waste, including bovine eyes and rooster combs^18^. Currently, this procedure is being replaced by production through bacterial fermentation^19,20^. These mentioned methods suffer from some major disadvantages, including possible contamination with the animal or bacterial toxins, expensive investment in scaling-up production systems, and the time-consuming processes of extraction and purification.

In recent years, there has been an increasing attempt to find an ideal bioreactor for HA production. The production of high-quality HA with low-cost and high-yield methods is the main challenge in the field of hyaluronan applied research.

Since the natural HA-producing organisms are mostly pathogenic, metabolic engineering currently represents an interesting opportunity to obtain HA from non-pathogenic, generally regarded as safe (GRAS) organisms.

The use of plant-based expression platforms offers some advantages for the production of recombinant human proteins compared to conventional expression systems such as bacterial, yeast, and animal cells. The particular benefits of the plant-based systems are high scale-up capacity, low purification costs, lack of harmful substances or endotoxins^21,22^, high stability of the engineered product, unique glycosylation patterns, and the capability of producing proteins with desired post-translational modifications. The first reports of the production of mammalian proteins in plants were performed in the late 1980s and known then on the concept of “plant molecular farming”^23–25^.

Compared to different plant-based expression systems, the hairy roots have received increasing attention as a versatile and efficient bioreactor to produce recombinant proteins^26,27^. The hairy root systems offer some advantages such as, high growth rate, easy culture, biological safety, low production costs, genetic and biochemical stability, and growth in defined media that are simple and free of hormones^28^. Hairy roots can do all Post-translational modifications that are important for human protein functions, such as stability, folding, secretion, and transport to the cell surface^25^. *A*. *rhizogenes*-mediated transformation which has been widely used in many plants, can introduce the desired gene into the plants after infection and subsequently induce hairy roots from the wounded surface of the explants^29,30^. The production of hairy roots in tobacco is preferable to other plants due to various factors such as access to tissue culture optimization protocols, ease of transformation, and high transgene expression^31^.

**Figure 1:**
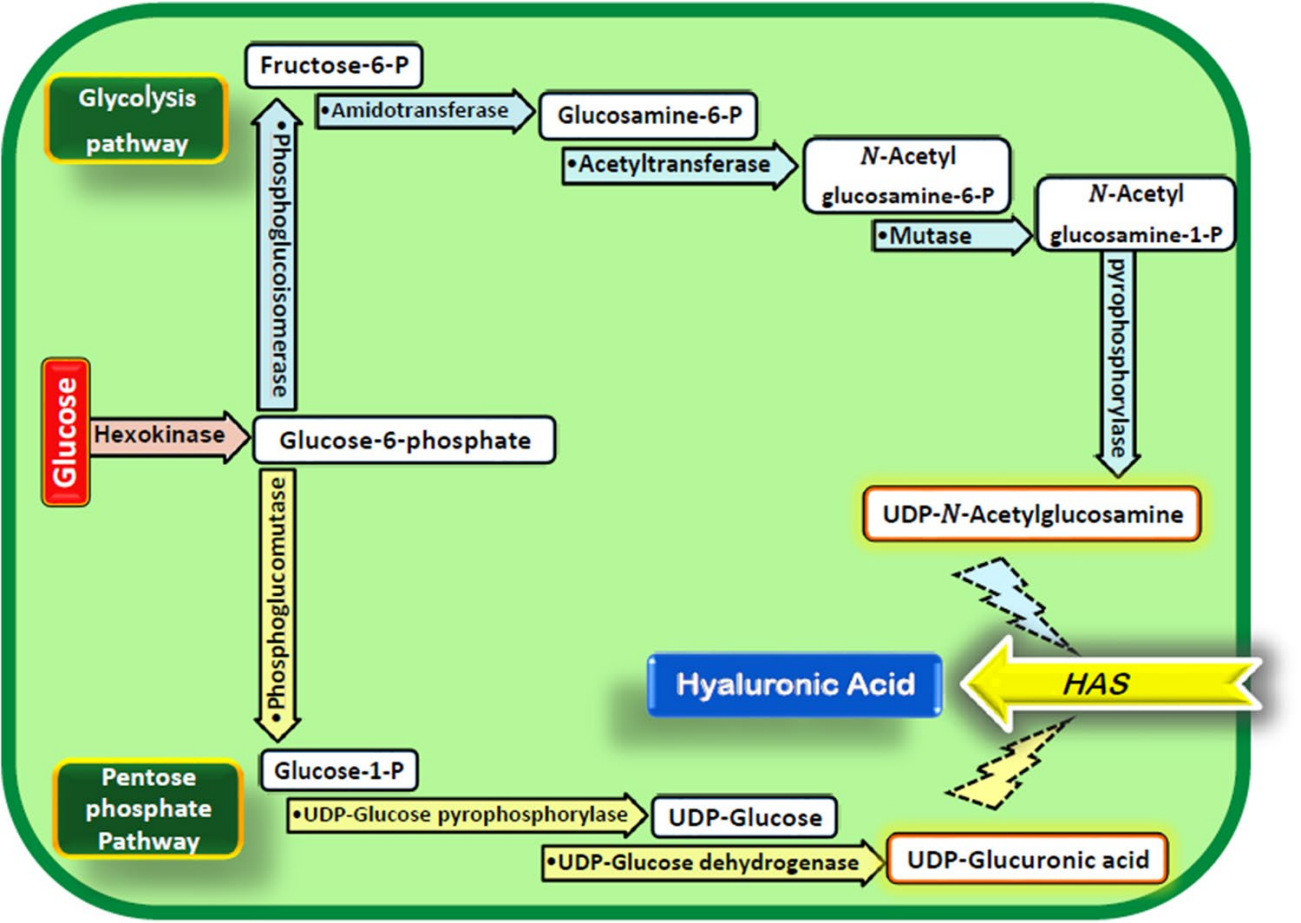
Schematic overview of the HA synthesis pathway in transgenic plants. HA precursors are produced in plants along the glycolysis and pentose phosphate pathways. By introducing only the *HAS* gene, plants are able to produce HA.

Hyaluronan is a biopolymer whose precursors (UDP-glucuronic acid and UDP-N-Acetylglucosamine) are produced in plants, along the glycolysis and pentose phosphate pathways^32^ (Fig. 1). Although plants are experts in the production of biopolymers such as cellulose and starch^33^, no HA is produced in plants due HAS deficiency^34^.

In this report, for the first time, the *hHAS2* gene was chosen for insertion in *Nicotiana tabacum* hairy roots. It was conclusively elucidated that rhHAS2 has a tremendous potential activity to synthesize a HMW-HA in tobacco hairy roots. Besides, a simple method for extracting HA from hairy roots was developed.

## Results

### Molecular analysis of transgenic hairy roots

About 10 days after the transformation of tobacco explants with recombinant *A. rhizogenes* containing the constructs pBI121-hHAS2, the roots began to develop and their growth continued in the selected medium. Roots were sub-cultured every 14 days by transferring one piece of root to the new selection medium. The purpose was the regeneration of transgenic hairy root clones and the elimination of agrobacterium contamination, respectively. Then, each transgenic hairy root line was transferred to the Erlenmeyer for further growth (Supplementary Fig. 3).

**Figure 2:**
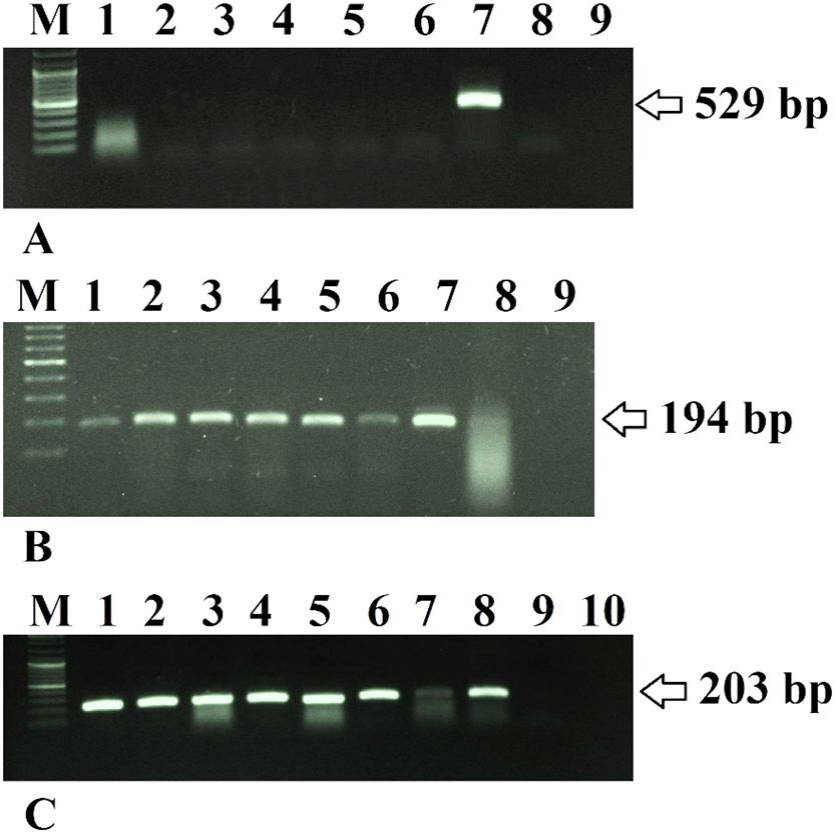
The absence of *A. rhizogenes* contamination was confirmed using PCR specific for *virG*. PCR amplification of a 194-bp fragment of *rolB* and a 203-bp fragment of *hHAS2* using DNA and cDNA derived from hairy roots. **(A)** Lane M: 100 bp DNA ladder Mix (Thermofisher, USA), lanes 1–6, no fragment was amplified using DNA template extracted from transgenic hairy root lines; lane 7, PCR amplification of the 529-bp fragment of *virG* using *A. rhizogenes* plasmid as a positive control; lane 8, non-transgenic hairy root; lane 9, water as a negative control. **(B)** Lanes 1–6, DNA template extracted from transgenic hairy root lines showing the amplification of a 194-bp fragment of *rolB*; lane 7, positive control (recombinant *A. rhizogenes* plasmid); lane 8, non-transgennic hairy root; lane 9, water as the negative control. **(C)** Lanes 1–7, cDNA template derived from transgenic hairy root lines showing the amplification of a 203-bp fragment of *hHAS2*; lane 8 positive control (recombinant *A. rhizogenes* plasmid) lane 9, water as a negative control; lane 10, cDNA template derived from a non-transgenic hairy root.

The putative transformants grown in the selection medium were first examined for the absence of *A. rhizogenes* contamination, by PCR using primers specific for *virG*, a bacterial gene that does not integrate into plant genomes. No specific fragment was amplified from these roots whereas, in *A. rhizogenes* as a positive control, the expected *virG* fragment was detected by PCR amplification of a 529-bp fragment (Fig. 2A). Hairy roots were rendered bacteria-free by transferring them weekly to a fresh medium. The presence of the *rolB* in the genome of hairy roots was confirmed by PCR amplification of a 194-bp fragment using specific primers (Fig. 2B). The *rolB* gene was revealed to be very efficient in promoting root formation. The 203-bp fragment belonging to *hHAS2* was amplified using cDNA templates extracted from the tobacco hairy roots using specific primer pairs. The presence of *hHAS2* was confirmed in selected lines (Fig. 2C).

Subsequently, PCR analysis of regenerated shoots with *hHAS2* specific primers revealed the presence of 203-bp fragment related to *hHAS2* gene in DNA and cDNA extracted from regenerated shoots.

Through the PCR, as performed by specific primers, it was confirmed that all hairy roots and regenerated shoots had been genetically transformed, and there was no sign of bacterial contamination in the transgenic lines.

### Assessment of rhHAS2 in transgenic hairy roots

The results confirmed the existence of the rhHAS2 in all transgenic hairy roots (Table 1). The highest rhHAS2 value of 65.72 ng/kg in transgenic hairy roots was measured by the human HAS2 ELISA kit.

**Table 1:** The rhHAS2 concentration in transgenic hairy roots**.**

Hairy roots	No. 1	No. 2	No. 3	No. 4	No. 5	NT
rhHAS2	62.31	65.72	49.4	60.8	54.6	0.08

Different levels of rhHAS2 were measured in all transgenic hairy root lines. Quantities are in ng/kg (wet weight).

*NT* non-transgenic.

### Assessment of HA in transgenic hairy roots

Evaluation of HA content in transgenic hairy root lines was performed using Carbazole, CTM (cetyltrimethylammonium bromide turbidimetric), and HA ELISA kit. The results showed a maximum concentration of 0.56 g/kg (wet weight) measured by HA ELISA kit (Table 2).

**Table 2:** Tukey pairwise comparisons of means (p < 0.05) were performed to compare the concentration of HA in transgenic hairy roots measured by Carbazole, CTM, and HA ELISA methods.

Hairy root lines	Carbazole	CTM	HA ELISA kit
No. 1	0.84 b	0.78 b	0.41 b
No. 2	1.35 a	1.12 a	0.56 a
No. 3	0.71 d	0.61 c	0.31 c
No. 4	0.78 c	0.73 b	0.39 b
No. 5	0.72 d	0.63 c	0.37 b
NT	0.07 e	0.06 d	0.006 d

Different letters followed by values in a column indicate significant differences at a 5% level of probability tested by Turkey’s test. Quantities are in g/kg (wet weight).

*NT* non-transgenic.

### Assessment of rhHAS2 and HA in regenerated shoots

The results confirmed the existence of the rhHAS2 enzyme in all regenerated shoots (Table 3). Evaluation of rhHAS2 concentration in regenerated shoots showed a maximum concentration of 40.8 ng/kg (wet weight). Assessment of HA in regenerated shoots was performed by the HA ELISA kit. The results showed a maximum concentration of 0.17 g/kg (wet weight) in the regenerated shoots (Table 4).

**Table 3:** Different levels of rhHAS2 were measured in all regenerated shoot lines.

Regenerated shoots	no.1	no.2	no.3	no.4	no.5	N.T
rhHAS2	40.31	42	28.4	40.8	37.6	0.05

Quantities are in ng/kg (wet weight).

*NT* non-transgenic.

**Table 4:** Tukey pairwise comparisons of means (p < 0.05) were performed to compare the concentration of HA in regenerated shoot lines measured by the HA ELISA kit.

Regenerated shoots	HA ELISA kit
No. 1	0.11 c
No. 2	0.17 a
No. 3	0.08 d
No. 4	0.13 b
No. 5	0.09 cd
NT	0.006 e

Different letters followed by values in a column indicate significant differences at a 5% level of probability tested by Turkey’s test. Quantities are in g/kg (wet weight).

*NT* non-transgenic.

### HA molecular weight determination

**Figure 3:**
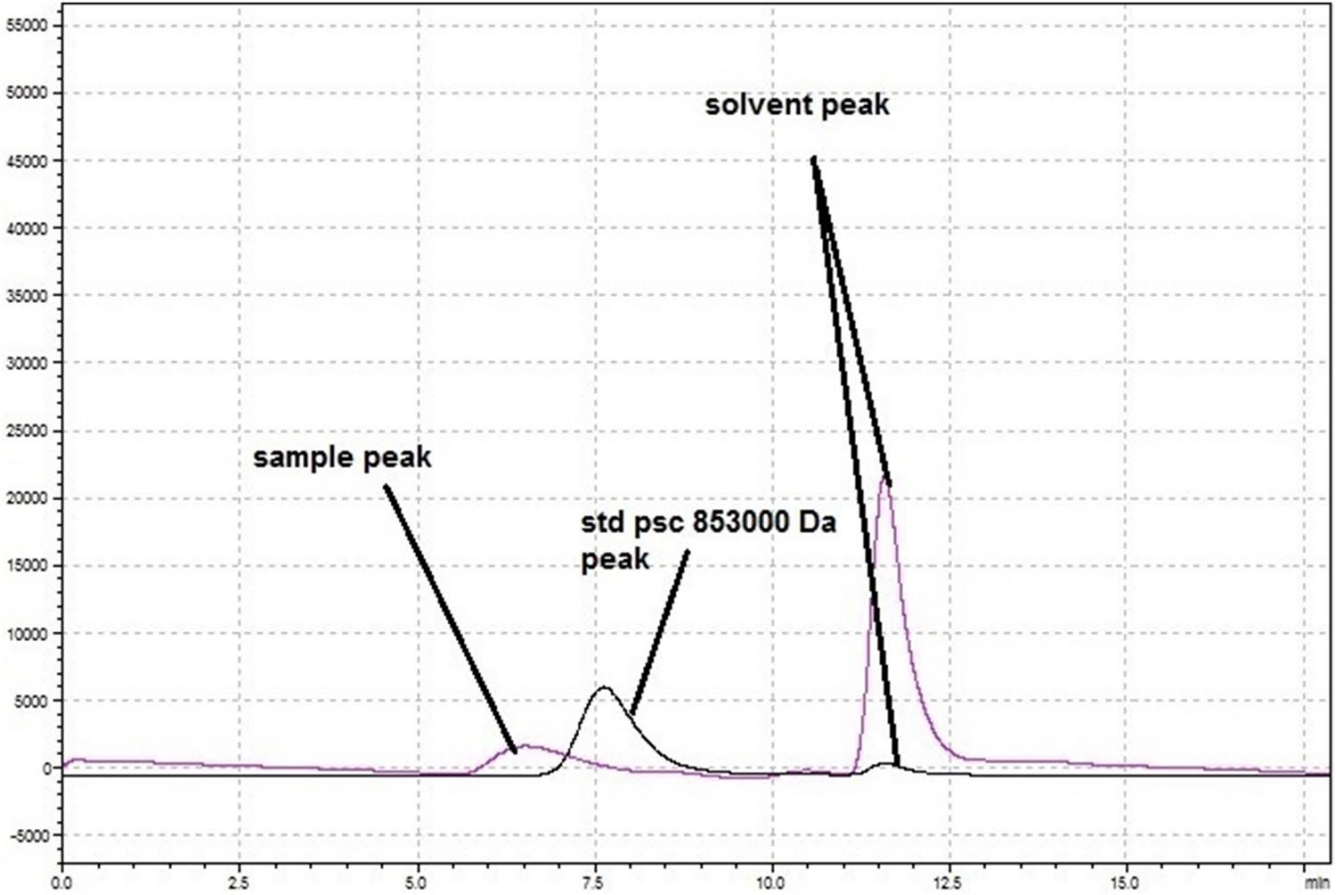
GPC analysis of the HA extracted from the transgenic hairy roots. The first pink peak showed HMW-HA > 853,000 Da.

The aqueous GPC analysis of HA extracted from transgenic hairy roots showed HMW-HA > 0.8 MDa. It is important to emphasize that the standard 0.8 MDa utilized in this study and any values above 0.8 MDa are considered high molecular weight HA (Fig. 3).

### Surface characteristics of HA Scaffolds in transgenic hairy roots

**Figure 4:**
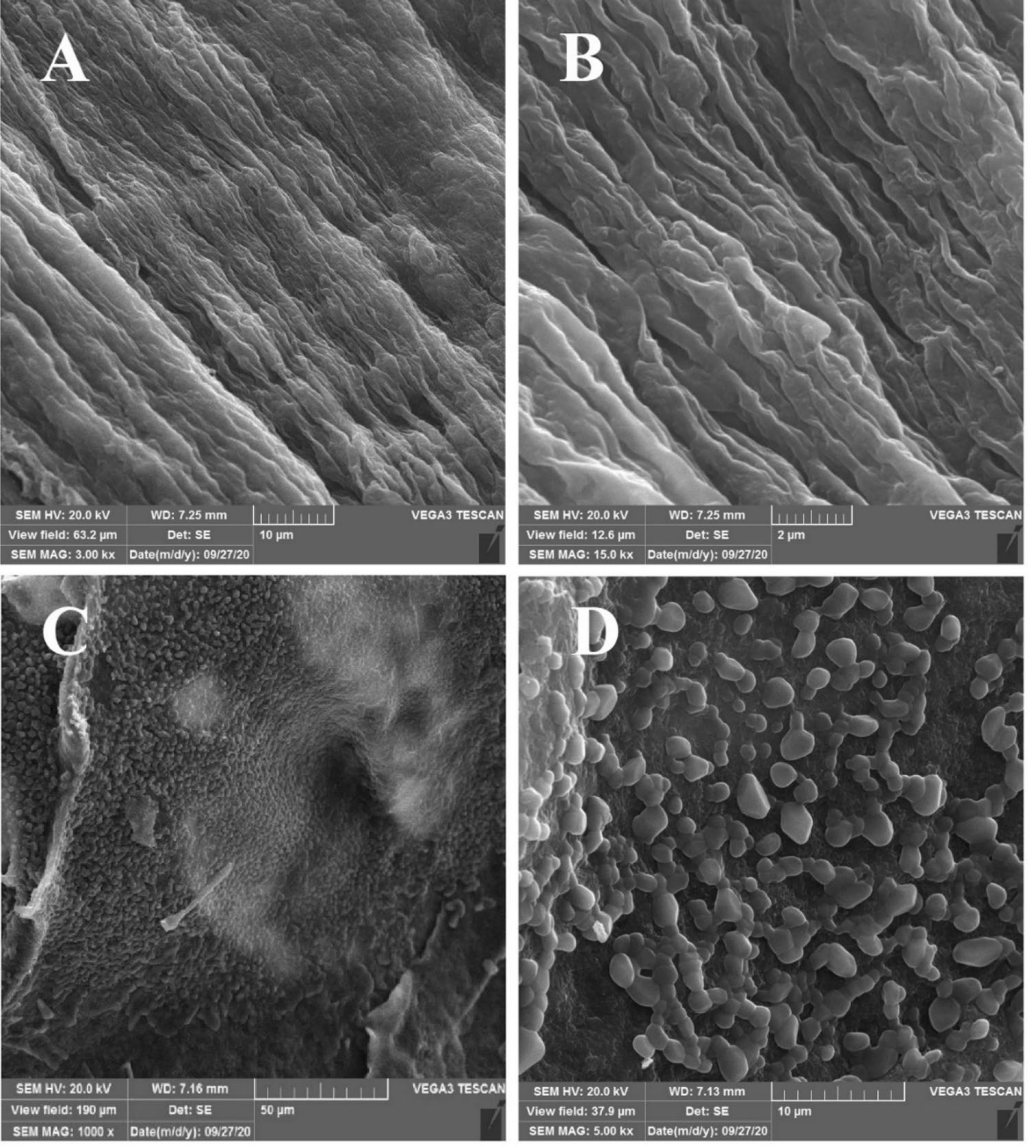
SEM analysis of non-transgenic and transgenic hairy roots. **(A,B)** Non-transgenic hairy roots with a wavy surface and free of any HA scaffolds on its surface. **(C,D)** SEM analysis of the transgenic hairy roots revealed a collection of irregular and small polygonal HA scaffolds on its surface.

Transgenic and non-transgenic hairy roots were analyzed using a scanning electron microscope. SEM analysis of non-transgenic hairy roots at high magnification imaging displayed a somewhat wavy surface architecture without any HA Scaffolds found on its surface. Microscopic analysis of transgenic hairy roots at high magnification showed small, irregular, and scattered polygonal aggregations patterns of the HA scaffolds on its surface. There were similarities between the observations in this study and that reported by Jong Hwan Kim et al., 2020 (Fig. 4)^35^. HA production in transgenic hairy roots was confirmed by electron microscopic analysis.

### DPPH radical scavenging activity

The antioxidant potential of transgenic and non-transgenic hairy roots was measured regarding their efficiency in scavenging free radicals generated by DPPH (1, 1-diphenyl-2-picryl-hydrazyl). Results revealed that the sample with the highest HA concentration of 0.56 g/kg (wet weight) exhibited an acceptable level of antioxidant activity of 46%. It should be noted that there was a small amount of DPPH radical scavenging related to the non-transgenic hairy roots, that was deleted from the DPPH radical scavenging of transgenic hairy roots.

## Discussion

The industrial production of HA has focused on a small number of production platforms based on the traditional fermenter-based expression systems^36^. In recent years, efforts to produce HA in heterologous systems have increased significantly. plant-based platforms for low-cost production of high-quality products, safety, and flexibility is well-known^23^. Hairy roots are a useful tool of plant biotechnology that effectively performs the majority of post-translational modifications required for the activity of eukaryotic proteins^25^. Glycosylation is one of the most common post-translational modifications necessary for the correct functioning of many proteins of human origin^37,38^. The trend of mammalian glycosylation is more similar to plant systems than other expression systems^37,39^. However, there are some differences between the recombinant glycoproteins that are produced in plants and animals^37^.

Many studies have shown that the high-level production of recombinant products in transgenic plants is related to the elements of the gene cassette, such as a strong promoter, proper polyadenylation site, and codon optimization between the target-gene sequence and the genome of the expression host^40^. The codon optimization of CDS based on the host expression system used plays an important role in heterologous gene expression^41^. The lack of codon optimization can reduce the level of gene expression. This is due to that the deficit of accessible tRNAs in the host, cause stopping the elongation of the target peptide or resulting in incomplete translation^30^. the most important factor altering the yield of recombinant proteins is subcellular targeting, which changes the folding, assembling, and PTM processes^37^. In the present study, the *hHAS2* CDS under the control of the strong CaMV 35S promoter was codon-optimized for expression in transgenic hairy roots. The KDEL tetrapeptide ER-retention signal was fused to the C-terminus of hHAS2 to target the recombinant protein to the ER lumen, which is a subcellular location that is protected from the host protease and contains a chaperone and glycosylation system for right folding and stability. The hHAS2 in transgenic hairy root cells can undergo a wide variety of post-translational modifications, which can regulate its activity for transport to the cell membrane and subsequently HA production.

**Figure 5:**
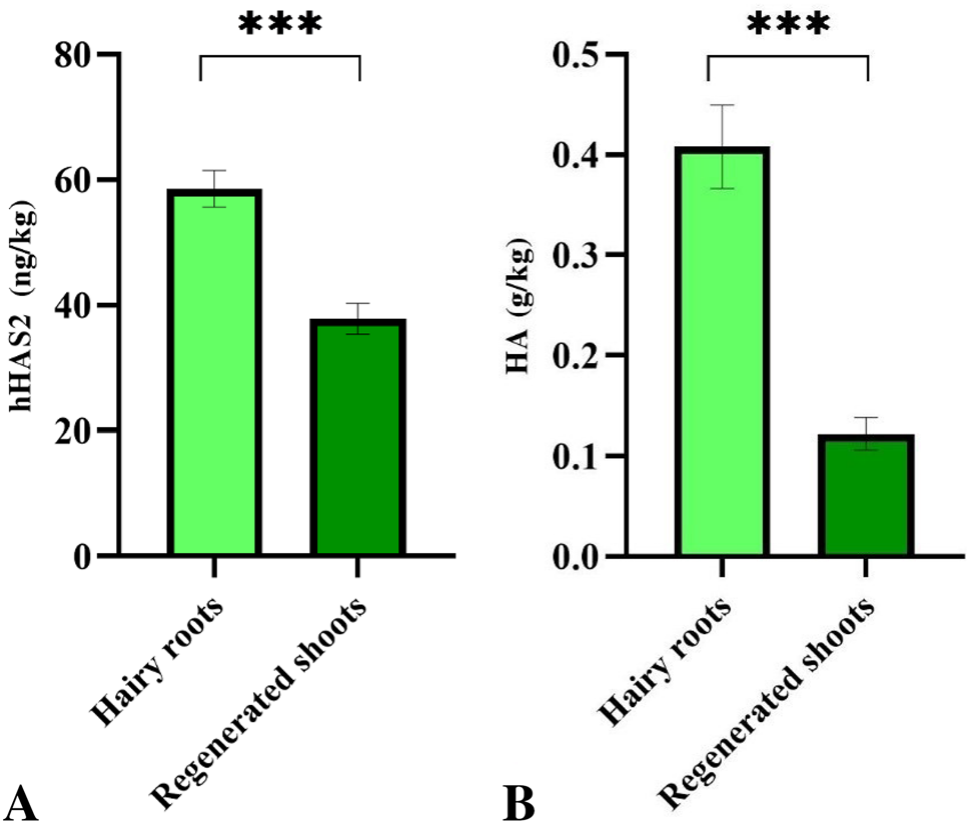
Comparison of hHAS2 and HA concentrations in transgenic hairy roots and the same regenerated shoots. **(A)** Comparison of hHAS2 concentration in hairy roots and regenerated shoots measured by hHAS2 ELISA kit. **(B)** Comparison of HA concentration in hairy roots and regenerated shoots measured by HA ELISA kit. The Student’s t-test was used for the hHAS2 and HA data. The values are expressed as mean ± standard error (SE) (***p < 0.001).

In a number of plant species, the transgenic hairy roots can spontaneously regenerate into whole plants^42,43^. In this study, transgenic hairy roots showed spontaneous regeneration of shoots on hormone-free both liquid and solid MS medium. The rhHAS2 was efficiently expressed in all transgenic hairy roots and the same regenerated shoots and then was measured by a human HAS2 ELISA KIT (Table 1). This kit is based on the biotin double antibody sandwich technology with high sensitivity and excellent specificity for the detection of hHAS2. According to the instruction of this kit, no significant cross-reactivity or interference between hHAS2 and other analogs was detected. The rhHAS2 concentration in transgenic hairy roots was significantly higher than regenerated shoots (Fig. 5A). The results, as shown in Tables 2 and 4, indicated acceptable rhHAS2 activity for HA production. The data reported here showed that the concentration of HA in transgenic hairy roots was significantly higher than regenerated shoots (Fig. 5B). It is accepted that the biggest advantage of hairy roots is that they often have a higher production capacity than other plant-based systems^44^. Hairy root systems have several advantages over whole plants, including high growth rates and the production of high levels of recombinant products^45,46^.

**Figure 6:**
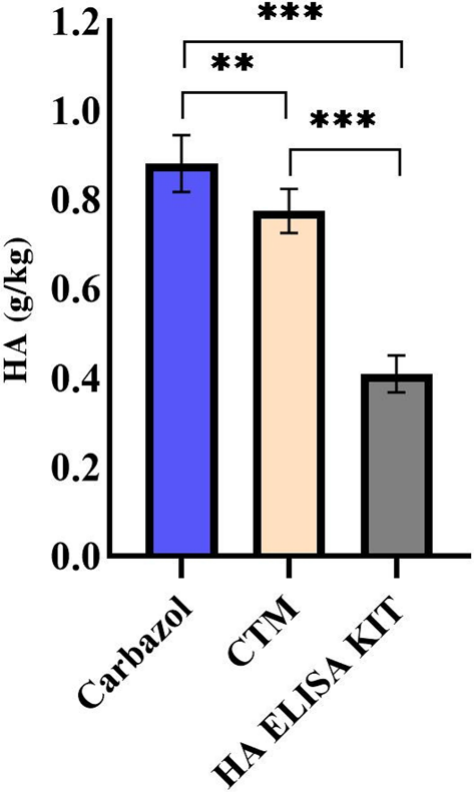
Comparison of HA concentration in transgenic hairy roots, measured by Carbazole, CTM, and HA ELISA kit. Quantities are in g/kg (wet weight). The Student’s t-test was used for the HA data. The values are expressed as mean ± standard error (SE). **p < 0.01, ***p < 0.001.

It can be seen from Table 5, the common methods for quantifying HA are Carbazole and CTM methods. Thus to compare the HA concentration in this study with previous studies, Carbazole and CTM methods were also used. As shown in Fig. 6, the mean values measured by Carbazole and CTM methods indicated a significant difference with the mean values measured by the HA ELISA kit.

**Table 5:** Engineered organisms for HA production**.**

Organism	*HAS* source	HA (g/l)	Molecular weight (MDa)	Quantification method	Ref.
*Enterococcus faecalis*	*S. pyogenes*	0.69	***	Carbazole	^53^
*Escherichia coli*	*S. pyogenes*	0.08	***	Carbazole	^53^
*Escherichia coli*	Human	***	***	***	^54^
*Bacillus subtilis*	*S. equisimilis*	0.81	1.2	Carbazole	^55^
*Lactococcus lactis*	*S. zooepidemicus*	0.65	***	HPLC	^56^
*Bacillus subtilis*	*S. zooepidemicus*	0.84	***	Carbazole	^57^
*Agrobacterium sp.*	*P. multocida*	0.3	0.7–2	Carbazole	^58^
*Escherichia coli*	*P. multocida*	0.55	1.5	Carbazole	^36^
*Lactococcus lactis*	*S. zooepidemicus*	0.59	0.88	Radioimmunoassay	^59^
*Lactococcus lactis*	*S. zooepidemicus*	0.11	2.8	Carbazole	^60^
*Tobacco Cells*	*Chlorovirus*	***	***	***	^61^
*Bacillus subtilis*	*P. multocida*	***	5.43	Carbazole	^62^
*Lactococcus lactis*	*S. zooepidemicus*	0.14	4.3	Carbazole	^63^
*Pichia pastoris*	*Xenopus laevis*	0.2	0.25	Carbazole	^64^
*Streptomyces albulus*	*S. zooepidemicus*	5.1	2	Carbazole	^51^
*Bacillus subtilis*	*S. zooepidemicus*	2.05	1.76	Carbazole	^50^
*Corynebacterium glutamicum*	*S. equisimilis*	0.14	***	CTM	^65^
*Corynebacterium glutamicum*	*S. equisimilis*	5.4	1.28	CTM	^49^
*Bacillus subtilis*	*S. equisimilis*	0.48	1.95	Carbazole	^66^
*Kluyveromyces lactis*	*P. multocida*	1.89	2.09	Carbazole	^10^
Tobacco hairy roots	Human	0.56	> 0.8	ELISA KIT	This study

***Information was not available.

These differences can be explained by the fact that the HA ELISA kit specificity is based on the use of proteins or proteoglycans that detect and bind to the HA and not to any other biological molecules. The major drawbacks to the Carbazole and CTM methods are the low specificity and the possibility of reacting with other polysaccharides in the tissue^47^. Furthermore, these methods are influenced by residual concentrations of salts and carbon sources present in the culture medium that are co-purified along with HA. However, Carbazole and CTM methods are less expensive and more available. Given that there is still reliable evidence that the CTM assay is more accurate than the Carbazole method^48^.

Among all organisms listed in Table 5, only four^10,49–51^ showed HA titers higher than 1 g/l. Therefore, the highest HA concentration of 0.56 g/kg (wet weight) measured by the HA ELISA kit, showed an acceptable hHAS2 activity for HA production in transgenic hairy roots. In this study, the transgenic hairy root lines showed slight phenotypic differences from non-transgenic hairy roots, which is an important factor for scaling^52^.

The molecular weight of hyaluronan from various sources is between 10^4^ and 10^7^ Da^67^. It has been demonstrated that the biological and physiological roles of HA are significantly dependent on its size^11^. For example in mammals, HMW-HA has a role in maintaining cell integrity, while low molecular weight HA (LMW-HA) is used as receptors and signaling agents^8^. As is well known, among hHAS isoforms, hHAS2 shows much higher enzyme activity and particularly is responsible for the synthesis of HMW-HA^68^. To determine the molecular weight of HA with the highest concentration of 0.56 g/kg (wet weight), aqueous GPC analysis was performed. It is important to emphasize that in GPC analysis there is an inverse relationship between molecular weight and retention time. As shown in Fig. 3, a standard with the molecular weight of 0.8 MDa was eluted out within retention of 7.7 min. Therefore, the first small pink peak with retention of 6.5 min, showed the existence of a high molecular weight substance > 0.8 MDa that was related to HA. The shortness of this peak was due to the low concentration of HA in the total volume of the sample solution. Because both the standard and the sample were dissolved in the same solvent, there were co-eluted peaks with similar retention of 11.5 min, which related to a low molecular weight of solvent.

One of the most disadvantages of HA extracted from bacterial or animal sources is the uncontrolled degradation of endogenous hyaluronidase-induced poly dispersion. The hyaluronidases (HYALs) are various groups of enzymes isolated from different origins such as vertebrates, leeches, and bacteria^34^. This enzyme specifically hydrolyzes the β-1,4 linkages of the HA molecule^35^. In general, the lack of endogenous HYAL in plant-based systems seems to lead to greater HA stability in such expression systems.

In previous studies, much attention has been focused on the unique appearance of HA as an antioxidant in pharmaceutical products^69,70^. DPPH is a stable free radical that significantly its absorbance decreases when exposed to the radical scavengers. In this study, in vitro antioxidant experiment of HA with the highest concentration of 0.56 g/kg (wet weight) showed the DPPH radical scavenging activity of 46%, which emphasizes the functional properties and health benefits of HA^71^. The ratio between N-acetyl-D-glucosamine and D-glucuronic acid has also been shown to influence HA molecular weight and concentration^72^. Thus, in this context overexpression of upstream genes that catalyze precursor preparation reactions could be effective.

## Conclusions

According to the present study, a rapid and efficient plant-based expression system has been developed to produce HA, which is not naturally produced in plants. Transgenic tobacco hairy roots provide a promising and safe system for HA production. Besides, HA accumulated in tubers, fruits, and roots is easily collected and can be used as food or for use in many cosmetic products, which in many cases reduces the cost of extraction and purification. Since HA production in transgenic plants can be provided at a low cost, current research is expected to expand rapidly in different industries.

## Materials and methods

### Ethical approval statements

This investigation is in accordance with relevant guidelines and regulations of Shiraz University. All experimental protocols were approved by the Institute of Biotechnology at Shiraz University.

### Construction of the pBI121-hHAS2 expression vector

The nucleotide sequences of *hHAS2* encoding hyaluronic acid synthase 2 (accession number: NM_005328.3) were obtained from the NCBI database (Supplementary Seq. 1). To designing of the gene construct, the Kozak sequence (acaaaatggc) and endoplasmic reticulum retention signal peptide KDEL (Lys–Asp–Glu–Leu) were added to the 5' and 3' end of the coding region of *hHAS2* respectively^24,52^. Besides, *Bam*HI (5′site) and *Sac*I (3′site) restriction sites were added for directional cloning into the same sites of the plant expression binary vector pBI121. Then, RNA destabilizing motifs and repeated sequences were dropped. Finally, the designed sequences were optimized based on the *N. tabacum* codon usage table using Gene Designer 2.0. The gene construct was chemically synthesized (General Biosystems, USA) and cloned into pUC57. Afterward, pUC57-hHAS2 recombinant construct was digested by the *Bam*HI and *Sac*I. Then, the *hHAS2* was cloned in the pBI121 vector and a recombinant pBI121–hHAS2 expression vector was designed. In this vector, which contains the *EPSPS* gene conferring resistance to glyphosate (5-enolpyruvylshikimate-3-phosphate), the expression of the *hHAS2* CDS was under the control of the CaMV 35S promoter and the nopaline synthase (NOS) terminator (Supplementary Fig. 1).

### Plant materials

Seeds of *N. tabacum* L. cv. Turkish were obtained from the Plant Virology Research Center, College of Agriculture, Shiraz University, Shiraz, Iran. The seeds were sterilized by being immersed in 70% (v/v) ethanol for 30 s. Afterward, they were soaked in 2% hypochlorite sodium for 10 min. Next, they were rinsed five times using sterile distilled water. Finally, the seeds were cultured in hormone-free MS medium solidified with 7 g/L plant agar with a pH 5.8 and autoclaved at 121 °C for 20 min^73^. The cultures were maintained at 25 °C for 2 weeks with a 16/8 h light/dark photoperiod until true leaves were just emerged and used as a source of explants for hairy roots induction.

### Induction and establishment of hairy roots

The aseptic leaf explants about 0.5–1 cm long were cut with a sterile scalpel and soaked in both transformed *A. rhizogenes* and non-transformed *A. rhizogenes* (as negative control) cultures with an OD_600 nm_ of 0.5 for 1 min. To remove the excess bacteria, the soaked explants were placed on sterile blotting paper. Next, the explants were cultured on an optimized co-cultivation medium (MS medium containing 100 µM acetosyringone solidified with 0.8% agar) under dark conditions at 25 °C^74^. After two days, explants were transferred and placed upside down onto MS medium supplemented with 30 mg/L meropenem and 0.5 mg/L glyphosates in the dark at 25 °C for 2 weeks^30^. Each hairy root developed at the marginal edges of the leaf fragments was maintained as a single line. Hairy roots were made bacteria-free by transferring to a fresh medium every two weeks, the hairy root lines were chopped into 3–4 cm pieces and sub-cultured on MS solid medium at the concentrations noted above and were incubated at 25 °C in the dark.

### Molecular analysis of hairy root lines

Total genomic DNA was extracted from transgenic and non-transgenic (negative control) hairy roots using a modified CTAB method^75^. Isolated DNA was used in PCR analysis for screening hairy roots. This was done by specific primers for *hHAS2* (oligonucleotide primers were designed using Allele ID 7 and Vector NTI 11 software and synthesized by MWG Biotech (Germany) (Table 6).

**Table 6:** Sequences Of the primers used for PCR-based characterization of the transgenic lines and the resulting product sizes.

Primers	Sequence	Product length (bp)	Ta (˚C)
*RolB*-F	AAGTGCTGAAGGAACAATC	194	54
*RolB*-R	CAAGTGAATGAACAAGGAAC		
*VirG*-F	CCTTGGGCGTCGTCATAC	529	55
*VirG*-R	TCGTCCTCGGTCGTTTCC		
*hHAS*2-F	AACATCTGGGCGAAAAACC	203	58.7
*hHAS2-*R	CGACATAAAGCGTAAGGAGCA		

The primers were designed using Vector NTI 11 and Allele ID 7 software.

*Ta* temperature annealing, *F* forward, *R* reverse.

The PCR mixture (20 μl) contained 7 μL of the master mix, 1 μL of each *hHAS2* specific primers (10 pmol), 10 μL of H_2_O, and 1 μL of genomic DNA (100 ng).

PCR for *hHAS2* was carried out by amplifying with an initial denaturation at 94 ºC for 5 min followed by 30 cycles of amplification with each cycle consisting of the following steps: 94 °C for 1 min, 58.7 °C for 30 s, and 72 °C for 45 s with a final extension of 72 °C for 10 min. The PCR products were analyzed by electrophoresis on 1% agarose gel in 0.5% TBE buffer.

To detect the transgenic nature of the hairy roots, PCR was performed using a set of *rolB* specific primers (Table 6) to amplify the 194 bp fragment. The PCR mixture and conditions were as described earlier. The PCR product was analyzed by 1% agarose gel electrophoresis.

Furthermore, primers that are specifically designed for the amplification of *virG* (Table 6) were used for confirming the elimination of infection by *A. rhizogenes.*

A 20 μl of PCR mixture contained 1 μl of each *virG* specific primers (10 pmol), 7 μL of the master mix, 10 μL of H_2_O, and 1 μL of genomic DNA (100 ng). The PCR conditions were 94 °C for 5 min followed by 30 cycles of amplification with each cycle consisting of the following steps: 94 °C for 30 s, 50.4 °C for 20 s, and 72 °C for 40 s with a final extension of 72 °C for 10 min. The PCR product was analyzed by 1% agarose gel electrophoresis.

Total RNA was extracted from transgenic hairy roots and non-transgenic ones using a Dena-zist RNA isolation kit (Tehran, Iran) according to the manufacturer’s instructions. The concentration of the RNA was measured using a Nanodrop device (Thermo Fisher Scientific, USA). The integrity and quality of the RNA were evaluated by visual observation of the 28S and 18S rRNA bands on a 1% agarose gel. Then, the extracted RNA was treated with RNase-free DNase (Thermofisher, USA). Afterward, 1 µg of DNase-treated RNA of each sample was used to synthesize the first strand of cDNA using oligo-dT primers according to the manufacturer’s instructions (Thermofisher, USA). The cDNA samples were stored at -20 °C until use. The expression of *hHAS2* was confirmed by *hHAS2* specific primers using cDNA of transgenic samples as a template.

The transgenic hairy roots were transferred to a 250 ml Erlenmeyer flask containing 30 ml of hormone-free MS liquid medium on an orbital shaker at 90 rpm in the dark at 28 ± 1 °C, for one month. The medium was refreshed weekly.

### Regeneration of whole plants from transgenic hairy roots

The amazing outcome achieved about each transgenic hairy root was their direct regeneration to seedlings after about 8 weeks, on hormone-free both liquid and solid MS medium as shown in Supplementary Fig. 5. These seedlings grew into fully-grown plants and developed flowers and seeds. Seedlings were replanted and maintained for 7 more days at 25 °C with a 16:8 h light–dark photoperiod. When true leaves turned green enough, plants were transferred to individual pots containing vermiculite and watered immediately in a climate-controlled glasshouse. Total genomic DNA and cDNA were extracted from regenerated shoots and non-transgenic plants (negative control). Isolated DNA and cDNA were used in PCR analysis for screening (Supplementary Fig. 6).

### Detection and quantification of hHAS2

The in vitro quantitative analysis of hHAS2 enzyme in transgenic lines was performed using the human HAS2 ELISA kit according to the manufacturer’s instructions (cat.No. E1578Hu/China). In the first step, transgenic lines were grounded using liquid nitrogen, Afterward, the powder (1 g wet weight) was suspended in (1:1) 0.1 M NaNO_3_ (w/v) and vortexed vigorously. Next, the cellular extracts were centrifuged at 7000 rpm for 20 min at 4 °C and then the supernatant was collected. According to the concentration of standards and the amount of optical density (OD), the linear regression equation of the standard curve was calculated (Supplementary Fig. 7). The concentration of hHAS2 in the samples was determined by comparing the OD of the samples to the standard curve.

### Extraction and pre-purification of HA

In the initial stage of the extraction, the transgenic lines were grounded using liquid nitrogen and the powder (1 g wet weight) was suspended in (1:1) 0.1 M NaNO_3_ (w/v) and vortexed vigorously for 2 min. Then, the suspensions were centrifuged at 7000 rpm for 20 min at 4 °C, and the supernatants were collected. After discarding cells, 1.5 volumes of ethanol were added to one volume of supernatant and the solution was kept at 4 °C for 1 h to enhance precipitation of HA. The suspensions were centrifuged at 7000 rpm for 10 min at 4 °C and the supernatant was discarded (Supplementary Fig. 8). Afterward, the precipitated HA was re-dissolved in 1 mL 0.1 M NaNO_3_ and kept at − 20 °C for further studies.

### HA quantification by ELISA kit

The HA concentrations in transgenic lines were quantified using the human HA ELISA kit (Cat.No. MBS262948/USA) according to the manufacturer’s instructions. The linear regression equation of the standard curve was calculated based on the concentration of the standards and OD value. Then, the HA concentration was assayed based on the standard calibration curve (Supplementary Fig. 11) and was multiplied by the dilution coefficient when calculating the results.

### Carbazole method

Carbazole-based quantification involves two steps: (1) hydrolyzing HA into glucuronic acid and glycosamine with 25 mM sodium tetraborate in sulfuric acid, and 2) coloring glucuronic acid with Carbazole. First, 200 µL of diluted samples )1:10( were introduced into glass tubes filled with 1 mL of 25 mM Na_2_B_4_O_7_ in H_2_SO_4_. The tubes were incubated at 100 °C for 15 min. After cooling on ice for 2 min, 40 µL of 0.125% (w/v) Carbazole in absolute ethanol was introduced to each tube and incubated at 100 °C for 15 min. After cooling, the absorbance was read at 550 nm^47^. A standard curve of HA concentrations ranging from 0.2 to 0.8 g/L was plotted using 99% HA (Sigma-Aldrich, St. Louis, MO, USA) (Supplementary Fig. 9). The HA concentration was calculated based on the standard calibration curve and the dilution ratio. Each sample was replicated three times and the average was considered the final result.

### CTM assay

In the first step, 200 µL of HA samples diluted 1:10 were introduced into 1.5 tubes filled with 200 µL of 0.1 M phosphate buffer pH 7. The tubes were incubated at 37 °C for 15 min. Then, 400 µL of CTM reagent (2.5 g CTAB dissolved in 100 mL of 2% (w/v) NaOH at 37 °C) was added to each tube and incubated for 10 min at 37 °C. Each tube was shaken for 10 s at the beginning and the end of this incubation. Absorbance was read at 600 nm against the blank (0.1 M phosphate buffer pH 7). The slope of the standard curve was obtained using linear regression in the HA concentration range between 0.2 and 0.8 g/L using 99% HA^47^ (Supplementary Fig. 10). The HA concentration was calculated based on the standard calibration curve and the dilution ratio. Each sample was replicated 3 times and the average was considered the final result.

### GPC analysis

The molecular weight of HA with the highest concentration was analyzed using the GPC method. As a solvent, a 0.1 M NaNO_3_ solution at the flow rate of 1 mL/min was utilized in a column system ultra-hydrogel linear (Shimadzu LC-20A). The injection volume was 20–50 μl with refractive index detectors. In GPC analysis a standard polysaccharide contained 853,000 Da was used.

### Scanning electron microscopy

Transgenic and non-transgenic hairy roots were analyzed for HA production using a scanning electron microscope (TESCAN-Vega 3, TESCAN, Czech Republic). The samples were first crushed using liquid nitrogen and suspended in (1:1) 0.1 M NaNO_3_ (w/v) then, were fixed in 2% (v/v) glutaraldehyde in phosphate buffer at 4 °C for 1.5 h. After that, the samples were dehydrated once in 50%, 75%, 90%, and twice in 100% ethanol series for 15 min for each step, respectively. After air-drying, the coverslip was coated with a sputtered gold coating and examined under a microscope.

### Antioxidant activity

The antioxidant activity of HA with the highest concentration was determined by a DPPH radical-scavenging assay^76^. An aliquot of the sample (100 μL) was mixed with 100 µL of 0.16 mM DPPH (Sigma-Aldrich Brazil Ltda) solution (in methanol). The mixture was shaken for 1 min then introduced into a 96-well microplate incubated at room temperature for 30 min in the dark. The absorbance was measured at 517 nm using a UV–visible spectrophotometer^76,77^. Radical scavenging activity (%) was calculated using the following equation:

$$\mathrm{Radical scavenging activity }\left(\mathrm{\%}\right)=\frac{\mathrm{A of control }-\mathrm{ A of sample}}{\mathrm{A of control }} \times 100.$$

### Statistical analyses

Comparisons between hairy root lines were caried out using the one-way analysis of variance and the Tukey HSD test. The level of statistical significance was set at p < 0.05. In addition, a two-tailed unpaired t-test was used to compare variables.

## Supplementary Information

Supplementary Information.

## Data Availability

All data generated or analysed during this study are included in this published article and its Supplementary Information Files. Extra data are available from the corresponding author upon request.
