# Supplementary material for: Heterologous production of hyaluronic acid in Nicotiana tabacum hairy roots expressing a human hyaluronan synthase 2
Source: Sci Rep. 2021 Sep 9;11:17966. doi: 10.1038/s41598-021-97139-0 (PMC8429445; doi:10.1038/s41598-021-97139-0)
Supplement: Supplementary file 1 — Supplementary Information. [file 41598_2021_97139_MOESM1_ESM.docx]

**Supplementary Material**

**Heterologous production of Hyaluronic Acid in *Nicotiana tabacum* hairy roots**

**expressing a human Hyaluronan synthase 2**

Arezoo Nazeri^1^, Ali Niazi^*1^, Alireza Afsharifar^2^, Seyed Mohsen Taghavi^3^, Ali Moghadam^1^, Farzaneh Aram^1^.

^1^ Institute of Biotechnology, Shiraz University, Shiraz, Iran.

^2^ Plant Virology Research Center, College of Agriculture, Shiraz University, Shiraz, Iran.

^3^ Department of Plant Protection, College of Agriculture, Shiraz University, Shiraz, Iran.

*Correspondence (Tel +987132272805; fax +987132272805; Email: [niazi@shirazu.ac.ir](mailto:niazi@shirazu.ac.ir))

**Supplementary sequencs 1**. Sequence of the *hHAS2* gene

*(accession number:* [*NM_005328.3*](https://www.ncbi.nlm.nih.gov/nuccore/NM_005328.3)*)*

|  | BamHI  GGATCCACAAAATGGCTAAAGCACTTACACTCGCGCTCTTTTTGGCACTATCACTCTATTTGCTGCCAAATCCCGCACACTCAATGCACTGCGAGAGGTTCCTATGTATCCTGCGTATAATCGGAACTACATTGTTTGGAGTGTCCCTTCTTTTGGGCATTACAGCTGCATATATCGTAGGGTATCAGTTTATCCAAACCGATAACTACTATTTCAGTTTTGGTTTGTACGGGGCTTTTCTTGCATCGCATCTTATTATCCAATCTCTTTTTGCATTTCTCGAACACAGGAAGATGAAAAAATCGCTTGAGACTCCCATCAAACTGAATAAGACCGTTGCTCTTTGCATCGCCGCGTACCAAGAAGATCCTGATTATCTCAGAAAATGCCTTCAGTCGGTTAAAAGGCTGACATACCCAGGAATCAAAGTCGTTATGGTCATTGATGGGAATTCAGAGGATGATCTTTATATGATGGACATTTTTAGCGAAGTTATGGGACGCGATAAGTCTGCCACTTATATCTGGAAAAACAACTTCCATGAGAAGGGGCCGGGTGAGACTGATGAATCTCATAAAGAAAGCTCACAGCACGTTACCCAATTAGTGCTCTCAAATAAGAGTATCTGTATCATGCAAAAATGGGGCGGCAAGAGAGAAGTGATGTACACCGCTTTTCGAGCTTTGGGCAGAAGTGTTGACTACGTGCAGGTTTGTGACTCTGACACGATGTTGGACCCCGCATCATCGGTGGAGATGGTTAAAGTGCTTGAGGAGGACCCTATGGTTGGTGGTGTAGGAGGCGACGTGCAAATCTTGAATAAGTATGATTCTTGGATTAGTTTCTTAAGTTCAGTTAGGTATTGGATGGCCTTTAACATTGAAAGGGCATGTCAATCATATTTCGGTTGTGTTCAATGTATTTCTGGGCCATTAGGCATGTACAGGAATTCTCTACTTCACGAATTTGTTGAAGATTGGTATAATCAAGAATTCATGGGAAATCAGTGTTCCTTTGGAGATGATCGCCACCTCACTAACAGAGTATTGTCACTAGGATATGCCACAAAGTACACAGCTCGGAGCAAATGCCTTACTGAAACACCGATCGAATACCTACGTTGGCTTAATCAGCAGACTCGATGGTCAAAATCCTACTTCCGCGAGTGGCTTTATAACGCTATGTGGTTTCATAAACATCATCTTTGGATGACATACGAAGCCATCATTACTGGTTTTTTCCCTTTCTTTTTAATAGCTACAGTTATTCAATTATTTTACAGAGGTAAGATTTGGAACATTCTCCTCTTTCTACTGACTGTGCAATTGGTTGGGCTTATCAAATCGAGTTTTGCGTCTTGTTTAAGAGGCAATATCGTCATGGTGTTCATGTCCCTCTATAGCGTCTTATACATGAGTTCGTTACTTCCTGCCAAAATGTTTGCTATTGCAACCATTAACAAGGCAGGATGGGGAACATCTGGGCGAAAAACCATTGTAGTTAATTTTATCGGCTTGATCCCTGTCTCAGTTTGGTTCACTATACTTCTTGGTGGAGTCATCTTCACCATCTATAAAGAATCAAAAAGACCTTTCTCCGAATCAAAGCAAACAGTTTTGATTGTCGGGACTCTTTTATACGCGTGTTACTGGGTGATGCTCCTTACGCTTTATGTCGTTCTCATCAACAAGTGTGGGAGAAGGAAGAAAGGACAACAATACGATATGGTTTTGGATGTCAAGGACGAGTTGCACCACCATCATCACCATTAAGAGCTC  SacI |
| --- | --- |


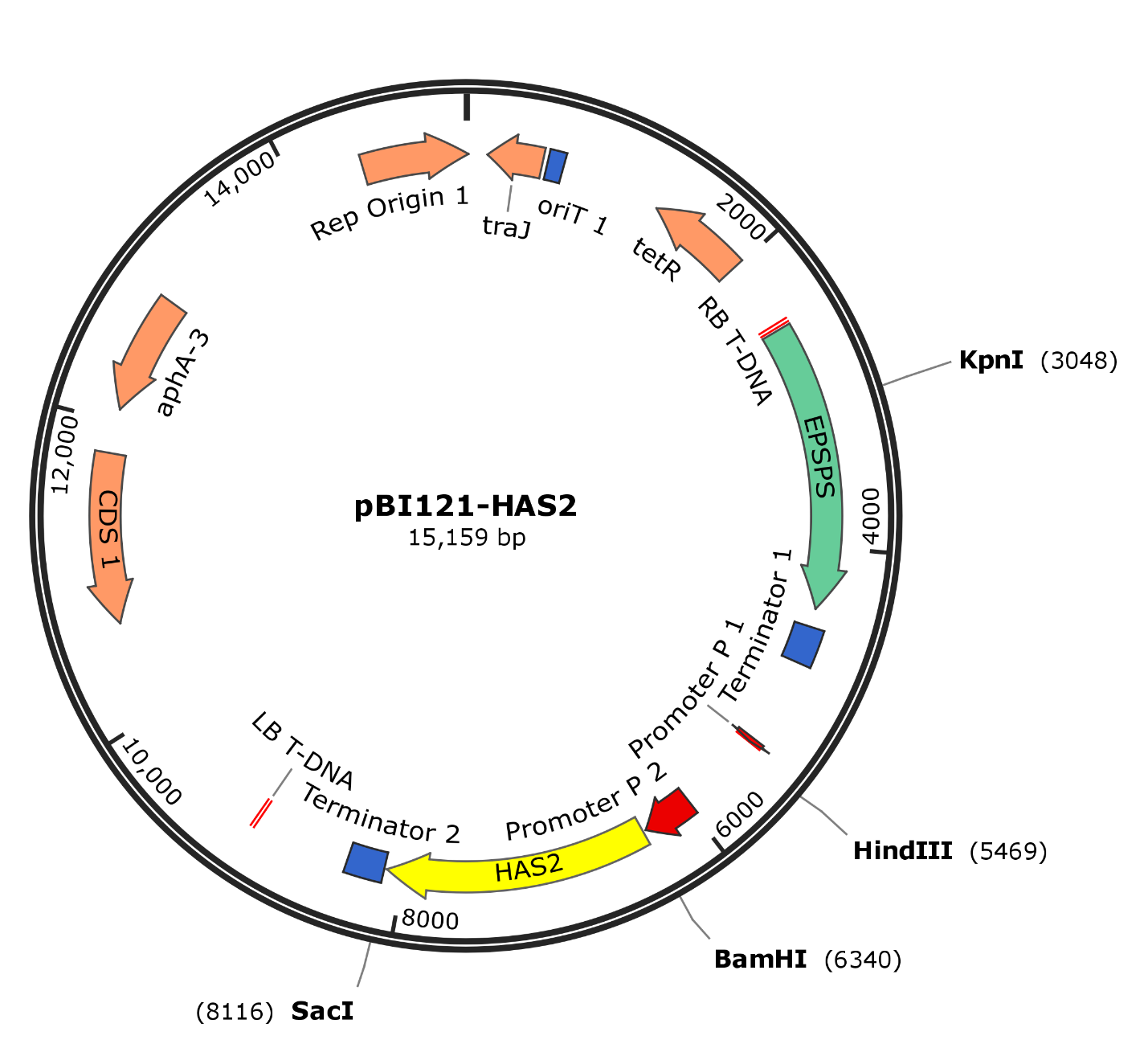


**Supplementary Figure 1.** The schematic map of the pBI121-hHAS2 expression vector. pBI121 expression vector contained the codon-optimized *hHAS2* gene under the control of the CaMV 35S promoter and a NOS terminator; EPSPS resistance gene; LB, T-DNA left border; RB, T-DNA right border and Restriction sites.


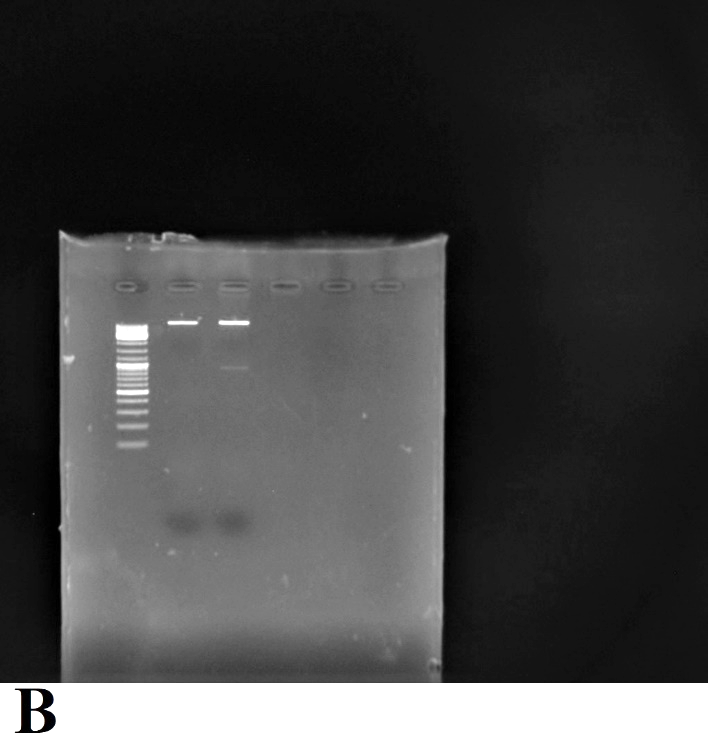

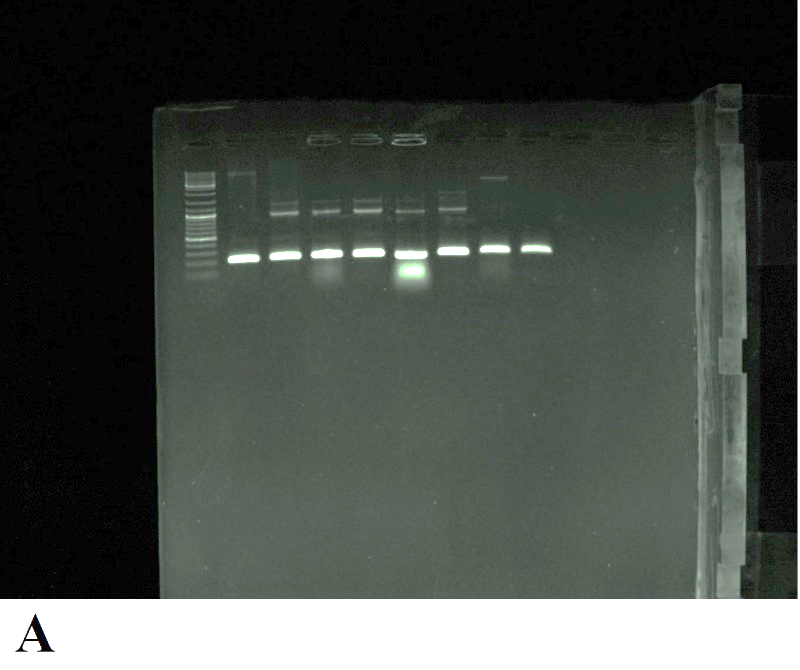
 **Supplementary Figure 2.** The presence of the construct harboring *hHAS2* in transformed *A. rhizogenes* was confirmed by PCR amplification of a 203-bp fragment and also digestion of transgenic recombinant plasmids using specific enzymes. (A) Lane M, Ruler 100bp DNA ladder Mix (Thermofisher, USA); lane 1, positive control (203-bp fragment from transformed *E. coli*); lane 2-8, transformed *A. rhizogenes* showed the amplification of 203-bp fragment; lane 9, non-transformed *A. rhizogenes* and lane 10, PCR negative control (water)**.** (B) Lane1, un-digested plasmids; Lane2, Digestion of recombinant *A. rhizogenes* plasmids by *Kpn*I and *Hin*dIII enzymes that released a 2421-bp fragment.

**
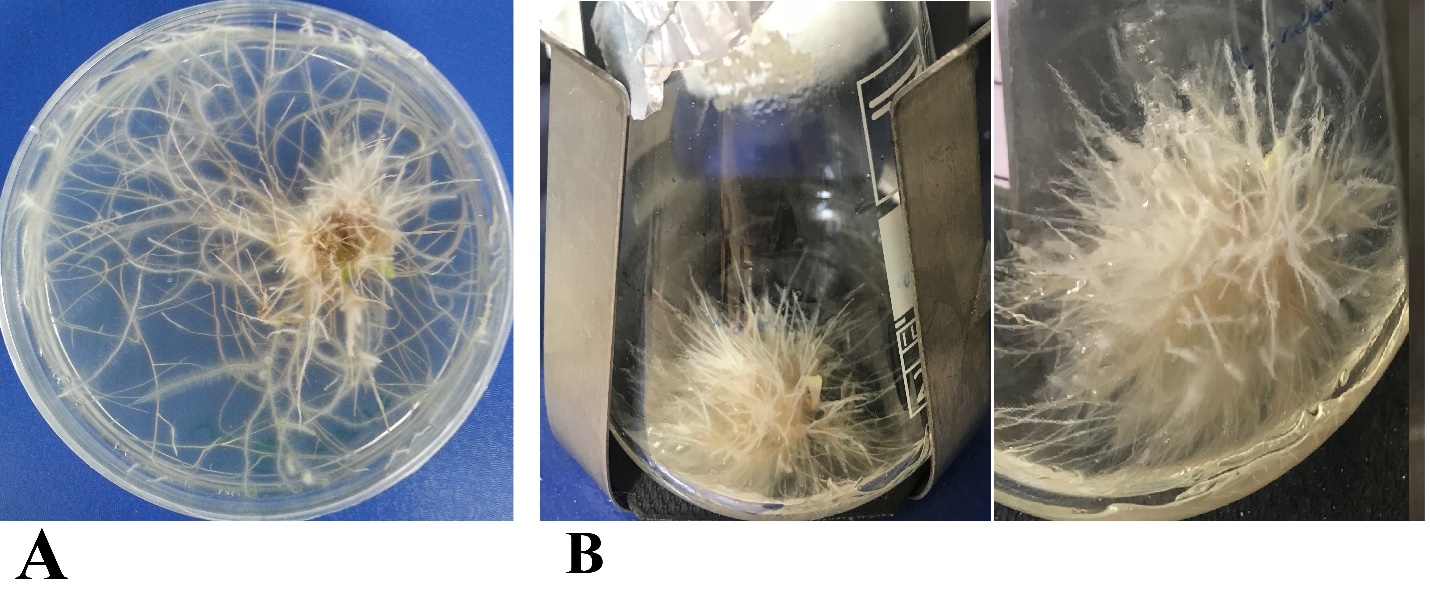
 Supplementary Figure 3.** Formation and elongation of *N. tabacum* hairy roots at different periods after *A. rhizogenes* infection. (A) Induction and propagation of hairy roots at the marginal edges of leaf explants grown on solid MS medium supplemented with 30 mg/L meropenem and 0.5 mg/L glyphosate at 25 °C under dark conditions after three weeks of post-infection. (B) Growth of hairy roots cultivated in a 250 mL Erlenmeyer flask containing MS liquid medium without antibiotics for one month at 28 °C in the dark with gentle shaking.


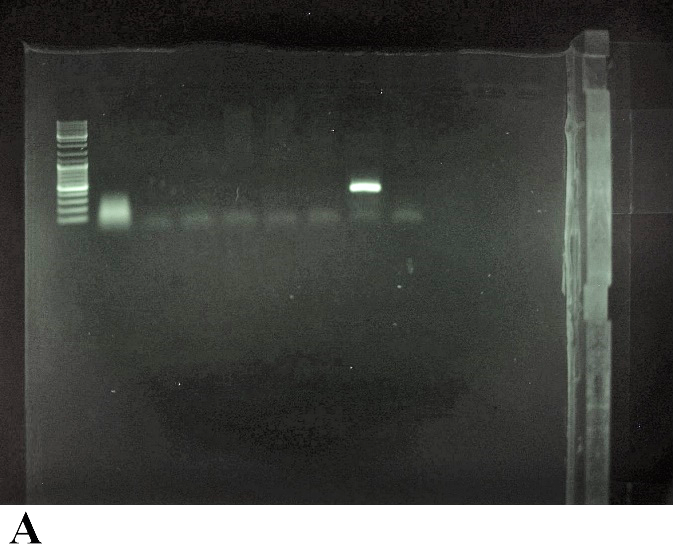

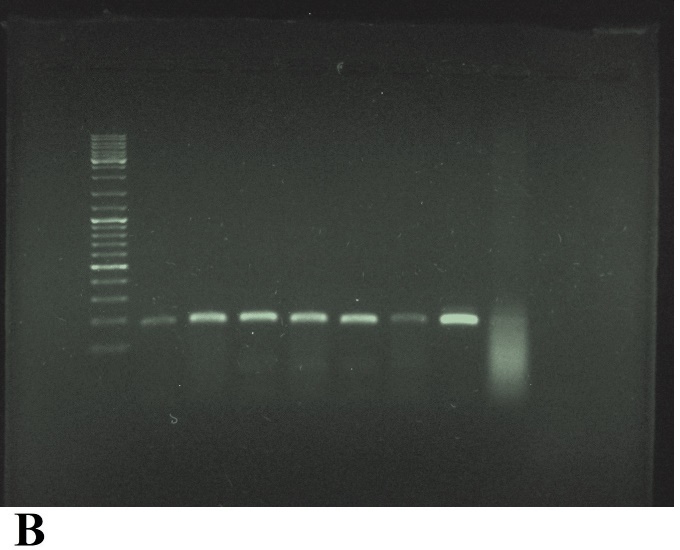


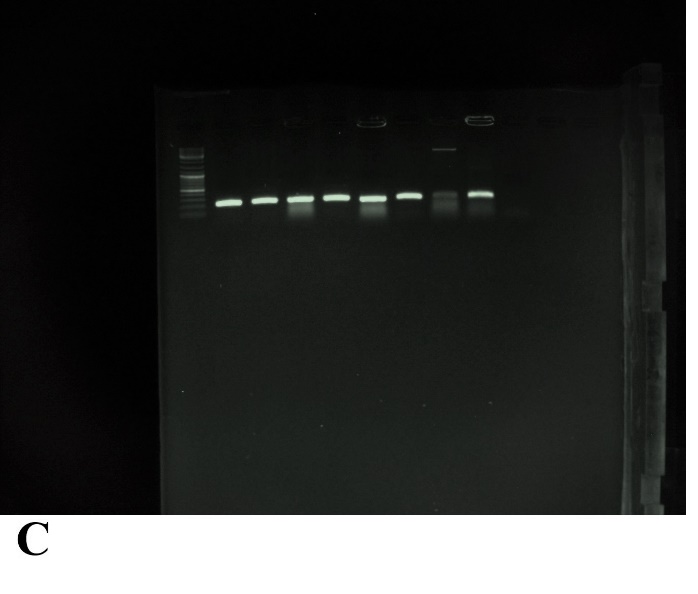


**Supplementary Figure 4.** The original gel-image of the results of PCR analysis. The absence of *A. rhizogenes* contamination was confirmed using PCR specific for *virG*. PCR amplification of a 194-bp fragment of *rolB* and a 203-bp fragment of *hHAS2* using DNA and cDNA derived from hairy roots. (A) Lane M: 100bp DNA ladder Mix (Thermofisher, USA), lanes 1-6, no fragment was amplified using DNA template extracted from transgenic hairy root lines; lane 7, PCR ampliﬁcation of the 529-bp fragment of *virG* using *A. rhizogenes* plasmid as a positive control; lane 8, non-transgenic hairy root; lane 9, water as a negative control. (B) lanes 1-6, DNA template extracted from transgenic hairy root lines showing the amplification of a 194-bp fragment of *rolB*; lane 7, positive control (recombinant *A. rhizogenes* plasmid); lane 8, non-transgennic hairy root; lane 9, water as the negative control. (C) lanes 1-7, cDNA template derived from transgenic hairy root lines showing the ampliﬁcation of a 203-bp fragment of *hHAS2*; lane 8 positive control (recombinant *A. rhizogenes* plasmid) lane 9, water as a negative control; lane 10, cDNA template derived from a non-transgenic hairy root.


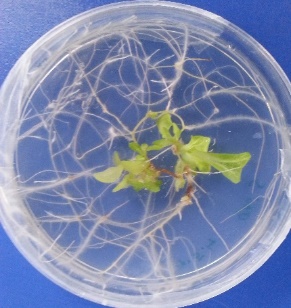

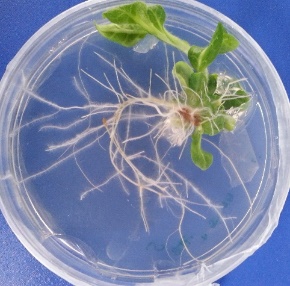

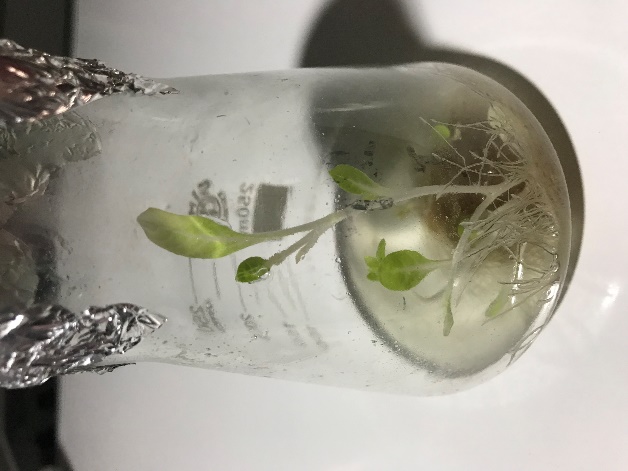

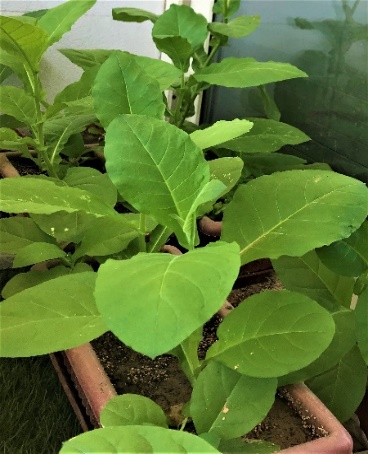


**Supplementary Figure 5.** Regeneration of whole plants from transgenic hairy roots. Shoot regeneration from transgenic hairy root lines on hormone-free liquid and solid MS medium containing 30 mg/L meropenem. After 8 weeks, adventitious shoots were regenerated spontaneously from the stock culture of positive hairy root lines.

**
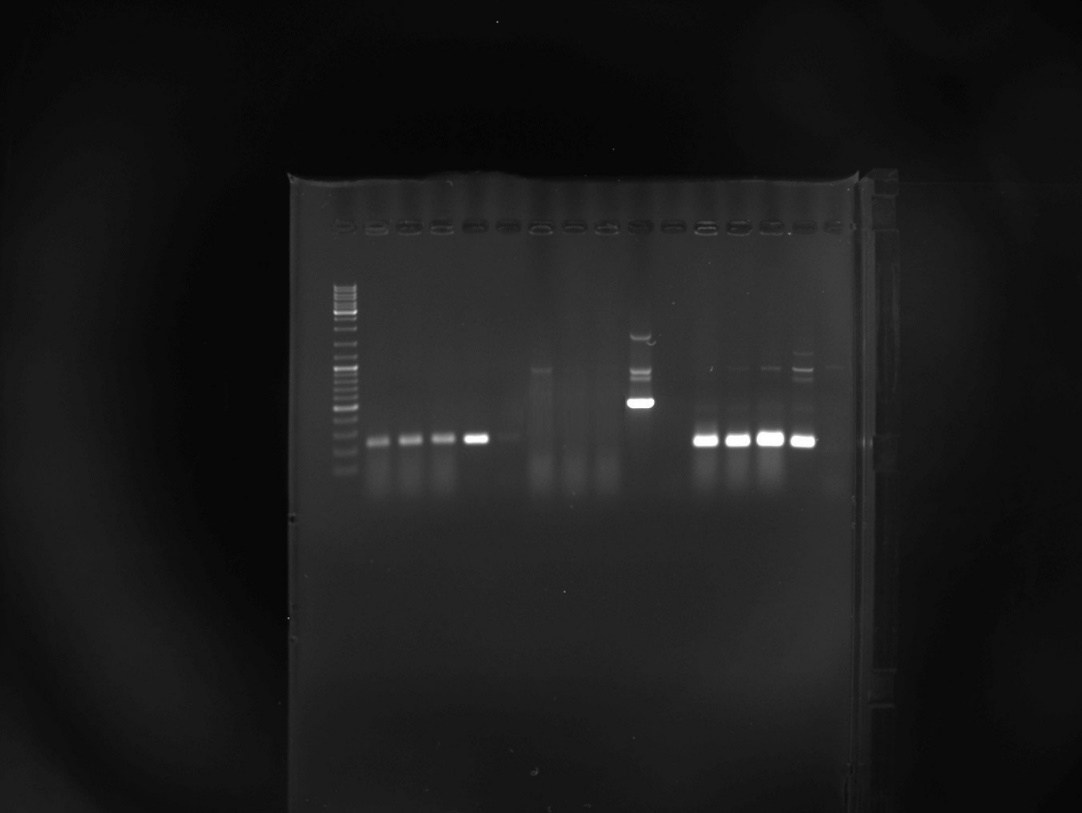
**

**Supplementary Figure 6.** Verification of integration of the *hHAS2* in regenerated shoots*.* Lane M: 100bp DNA ladder Mix (Thermofisher, USA); lanes 1-3: cDNA template derived from regenerated shoot lines showing the ampliﬁcation of a 203-bp fragment of *hHAS2*;lane 4 positive control (recombinant *A. rhizogenes* plasmid); lane 5, DNA templates derived from wild-type plant; lane 6-8 no fragment was amplified using DNA template extracted from transgenic hairy root lines; lane 9, PCR ampliﬁcation of the 529-bp fragment of *virG* using *A. rhizogenes* plasmid as a positive controllane 10, non-transgenic hairy root; lanes 10-12, DNA template extracted from transgenic hairy root lines showing the amplification of a 194-bp fragment of *rolB*; lane 13, positive control (recombinant *A. rhizogenes* plasmid); lane 14, non-transgennic hairy root.

**
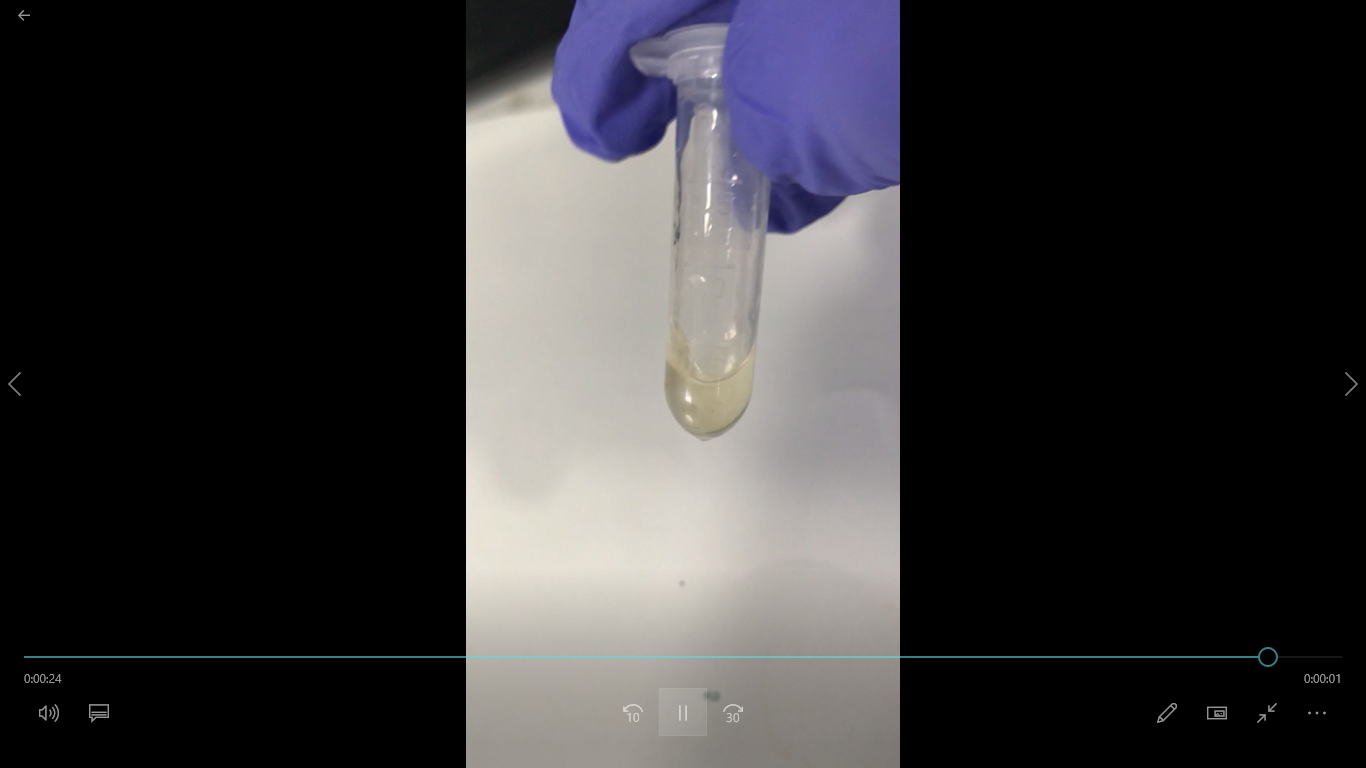
**

**Supplementary Figure 7.** Collecting the HA using ethanol precipitation.

**Supplementary Figure 8.** The standard curve for human HAS2 ELISA KIT.

**Supplementary Figure 9.** The standard curve for carbazole method constructed in this study and utilized for HA quantification. Experiment realized in Triplicate.

**Supplementary Figure 10.** The standard curve for CTM method constructed in this study and utilized for HA quantification. Experiment realized in Triplicate.

**Supplementary Figure 11.** The standard curve for HA ELISA KIT.

**Supplementary video 1.** HA extracted from transgenic hairy roots.
